# Early Miocene elevation in northern Tibet estimated by palaeobotanical evidence

**DOI:** 10.1038/srep10379

**Published:** 2015-05-15

**Authors:** Bin Sun, Yu-Fei Wang, Cheng-Sen Li, Jian Yang, Jin-Feng Li, Ye-Liang Li, Tao Deng, Shi-Qi Wang, Min Zhao, Robert A. Spicer, David K. Ferguson, Rakesh C. Mehrotra

**Affiliations:** 1 State Key Laboratory of Systematic and Evolutionary Botany, Institute of Botany, Chinese Academy of Sciences, Beijing 100093, China; 2 Key Laboratory of Vertebrate Evolution and Human Origins, Institute of Vertebrate Palaeontology and Palaeoanthropology, Chinese Academy of Sciences, Beijing 100044, China; 3 Environment, Earth and Ecosystems, Centre for Earth, Planetary, Space and Astronomical Research, The Open University, Milton Keynes, MK7 6AA, UK; 4 Department of Palaeontology, University of Vienna, Althanstrasse 14, A-1090 Vienna, Austria; 5 Birbal Sahni Institute of Palaeobotany, 53 University Road, Lucknow 226007, India; 6 Henan University of Traditional Chinese Medicine, No.1 Jinshui Road, Zhengzhou, Henan, 450008, China; 7 University of Chinese Academy of Sciences, Beijing 100039, China

## Abstract

The area and elevation of the Tibetan Plateau over time has directly affected Asia’s topography, the characteristics of the Asian monsoon, and modified global climate, but in ways that are poorly understood. Charting the uplift history is crucial for understanding the mechanisms that link elevation and climate irrespective of time and place. While some palaeoelevation data are available for southern and central Tibet, clues to the uplift history of northern Tibet remain sparse and largely circumstantial. Leaf fossils are extremely rare in Tibet but here we report a newly discovered early Miocene barberry (*Berberis*) from Wudaoliang in the Hoh-Xil Basin in northern Tibet, at a present altitude of 4611 ± 9 m. Considering the fossil and its nearest living species probably occupied a similar or identical environmental niche, the palaeoelevation of the fossil locality, corrected for Miocene global temperature difference, is estimated to have been between 1395 and 2931 m, which means this basin has been uplifted ~2–3 km in the last 17 million years. Our findings contradict hypotheses that suggest northern Tibet had reached or exceeded its present elevation prior to the Miocene.

Despite the critical role Tibet plays in understanding the processes linking subcrustal dynamics to climate decades of research have failed to produce a clear view of the plateau’s elevation history^1^,^2^. Data evidencing the elevation of different parts of Tibet over time have been mainly divided into two types^3^. One represents an indirect analysis^2^,^3^,^4^, such as crustal movement models based on geomorphological evidence or rates of sedimentation. The other one constitutes a direct quantitative estimation of Tibet’s palaeoaltimetry, mostly based on δ^18^O values in carbonate sediments^5^,^6^,^7^,^8^,^9^,^10^, but with two case studies utilizing the climate leaf analysis multivariate program (CLAMP)^11^,^12^.

Current opinions consider that south central Tibet reached or exceeded its current elevation by the Miocene, and since then there has been minimal uplift, or possibly even a 1 km deflation^5^,^6^,^12^. By 11 Ma (late Miocene) the Thakkhola Graben appears to have reached a height of 4500 ± 430 m to 6300 ± 330 m or 3800 ± 480 m to 5900 ± 350 m^6^, between 8 Ma and 2 Ma (the late Miocene to Pliocene) Gyirong was elevated to 5850 + 1410/−730 m^7^, and the Zanda Basin rose to between 4000–4500 m since 9.2*–*1 Ma^8^,^13^. In central Tibet, the Lunpola Basin reached about 4000 m by 35 ± 5 Ma^9^, and the Nima Basin had an elevation of 4500–5000 m at 26 Ma^10^.

Interpreting the history of northern Tibet Plateau’s uplift generates controversy due to the paucity of palaeoaltimetric data and a lack of consensus. For example quantitative estimations of palaeoaltimetry for the northern Tibetan Plateau, correspondingly, are also divided with estimates for the height of the Hoh-Xil Basin ranging from ~2000 m to 4000 m at 39*–*36 Ma^14^,^15^,^16^, while modelling studies suggest that either there has been a significant uplift of northern Tibet since the Miocene (~15 Ma)^2^,^4^, or that it was near or above its modern elevation at 20 Ma^17^.

Different methods used for estimating the palaeo-elevations of the Tibetan Plateau have their own advantages and disadvantages, which means cross validation is needed in order to overcome their innate shortcomings.

Palaeoelevation estimations based on biological evidence differ from those using geophysical methods in that the discoveries of biological evidence (fossil materials) are usually chance occurrences and rare. Nevertheless, the unique advantage of biological evidence is that fossils may be clearly identified to a particular taxon and if that taxon is extant its unique ecological niche can be used to infer past conditions, including palaeoelevation.

Here we report a fossil *Berberis* leaf from the early-middle Miocene sediments of the Wudaoliang Basin in northern Tibet, recovered from a present day altitude of 4600 m, whereas its living modern counterpart (*B. asiatica*) is confined to altitudes of 914*–*2450 m. It provides an opportunity to estimate the palaeoaltimetry of the Wudaoliang Basin during the early Miocene and to evaluate previous hypotheses regarding the uplift of northern Tibet.

## Material

### Geological context

**Figure 1:**
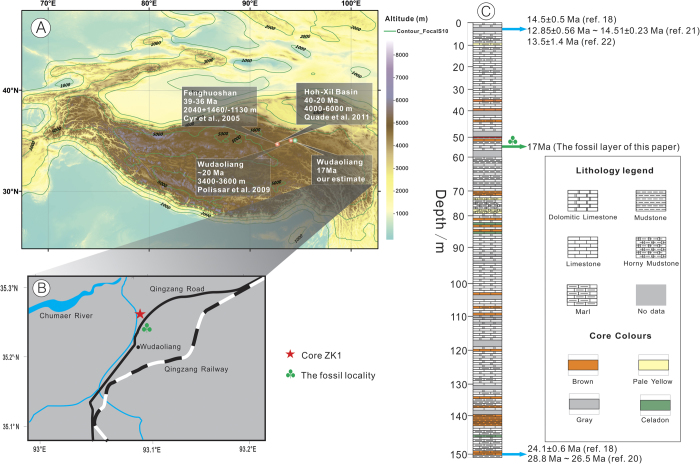
Map showing the fossil locality (A, B) and the stratigraphical section of the Wudaoliang Group (C). A showing the Tibetan Plateau and quantitative estimations of palaeoaltimetry for northern Tibet. B showing the locations of our fossil (35°13’56.90“N, 93° 05'10.50“E, 4611 ± 9 m) and core ZK1 (35°14’18.40“N, 93° 05'22.80“E, 4666 m). C showing the stratigraphical section of core ZK1^18^, including the ages at top and bottom of the Wudaoliang Group, which are in accordance with previous data from dating of the Wudaoliang volcanics^20^,^21^,^22^. The maps are created by authors using “ArcGIS 10” and “CorelDraw 14” software. The stratigraphical section is drawn by authors using “CorelDraw 14” software based on the data from refs. 18, 20, 21, 22.

The solitary fossil leaf specimen that we can identify accurately was collected from a natural outcrop of lacustrine sediments of the Wudaoliang Group near Wudaoliang Town, Qinghai Province, northern Tibetan Plateau (Fig. 1A,B, 35°13’56.90“N, 93° 05'10.50“E, 4611 ± 9 m). The fossil leaf is preserved as an impression on a matrix of pale-yellow, fine-grained marl and is now housed in the Institute of Botany, Chinese Academy of Sciences in Beijing (specimen no. WDL 2010-001).

The same block contains another specimen (see: SI, Figure S2) and numerous other specimens occur in the adjoining layers (see: SI, Figure S3). Most specimens were not well enough preserved to allow us to pin-point their systematic position but one fossil leaf of *Berberis* was sufficiently well preserved to allow its determination to species level and provide a palaeoelevation datum.

### Age estimates for the fossil

In the study area, the ZK1 drill hole (see SI for core description), 720 m away from the fossil locality, penetrated the Wudaoliang Group to a depth of 154 m^18^. The dating of core ZK1 (Fig. 1) is calibrated using climatostratigraphic methods, based on a comparison between the palaeoclimatic cycles recorded by carbon and oxygen isotope changes within lacustrine deposits^18^ and the palaeoclimatic cycles recorded by the deep-sea oxygen isotope curve^19^. The ages at both top and bottom of the Wudaoliang Group calibrated in this way are in accordance with those from dating of Wudaoliang volcanics^20^,^21^,^22^ (see more details in SI). Based on carbon and oxygen isotope studies, the age of these lacustrine sediments is regarded as early to middle Miocene (24.1*–*14.5 Ma)^18^. Comparisons of the coordinates and altitude of our fossil layer with those of core ZK1, enabled us to correlate the fossil layer to a depth of 55 m in the ZK1 drill hole, hence an age of ca. 17 Ma. (Fig. 1C, see more details in SI).

## Result

### Systematic treatment

The detailed fossil description and taxonomic discussions are included in SI. The specific combination of leaf characters in the fossil such as its dentate margin with 4-5 fine spinose or setaceous teeth on each side, pinnate festooned brochidodromous venation, the presence of inter-secondary veins and random reticulate tertiary veins falls within the circumscription of the genus *Berberis* of the Berberidaceae^23^.

By eliminating the obviously different species in the genus (see the detailed elimination in SI) we were left with only 4 most similar species. The supplementary tables and keys (see SI, Table S1 – S2, Key S1 – S2) clearly show that two, or more than two, morphological character differences exist between our fossil specimen and the other 3 extant species except *B. asiatica*. A survey of herbarium material of *Berberis* revealed that these leaf architectural characters are stable at the species level and provide a robust basis for identification at the generic and species levels^24^.

**Figure 2:**
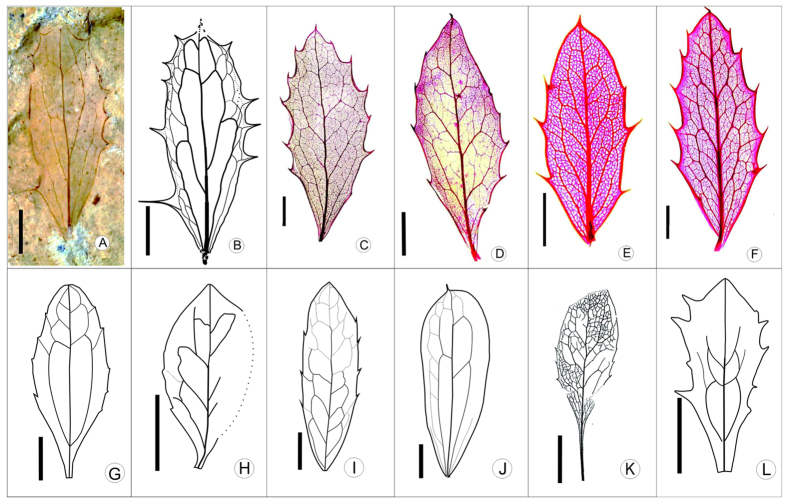
Leaf architecture of extant (C–F) and fossil (A, B, G–L) *Berberis*. **A, B.** Fossil from Wudaoliang, C. *B. asiatica*, D. *B. chitria*, E. *B. taronensis*, F. *B. phanera*, G. *B. huziokai*, H. *B. teutonica*, I. *B. lycium*, J. *B. poblana*, K. *B. longipetiolata*, L. *B. ahuehuetensis* A, B, D, E, F, scale bar = 0.5 cm. C, G, H, I, J, K, L, scale bar = 1 cm. The photographs of fossil specimen (A) and extant *Berberis* (C–F) are taken by authors., the leaf architecture drawing (B) is drawn by authors using “CorelDraw 14” software, and drawings (G–L) are cited from Ref. 23.

The detailed comparison with fossil and extant species of *Berberis* (see SI for comparison, Fig. 2) indicates that the fossil cannot be distinguished from extant *B. asiatica*, for the leaf architectural characters of the fossil are identical to those of *B. asiatica* except that no veinlet are preserved in the fossil. If it were not for the fact that the specimen is a fossil, it would be unequivocally assigned to *B*. *asiatica* (Fig. 2C). However, considering its fossil state it is referred to *B*. cf. *asiatica*.

**Figure 3:**
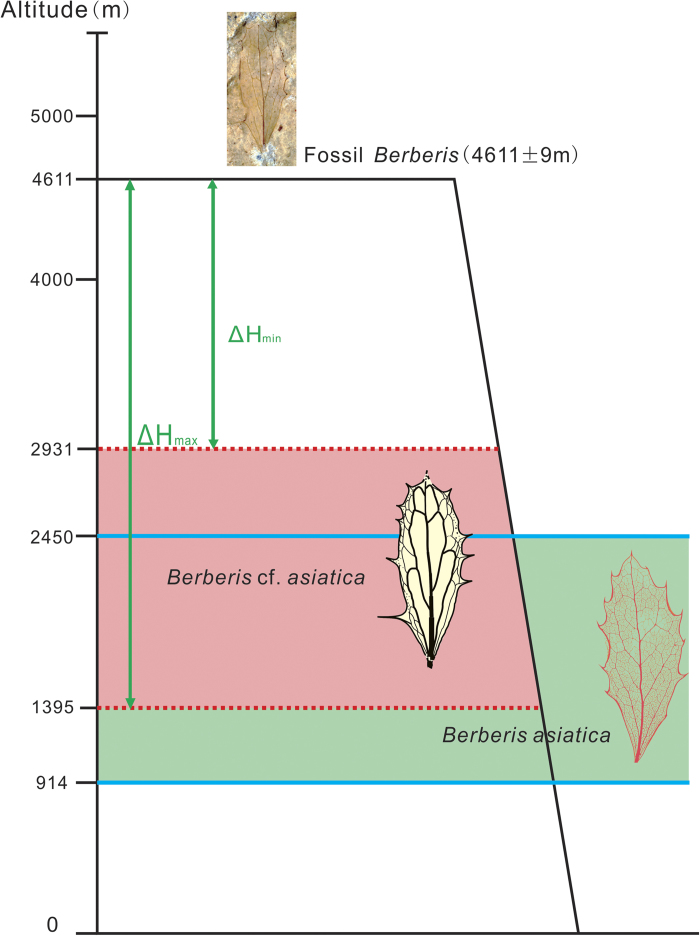
The uplift of fossil locality. Green range: altitudinal range of *B. asiatica*, Pink range: altitudinal range of *B.* cf. *asiatica*. ΔH_min_: the minimum value of uplift, ΔH_max_: the maximum value of uplift. This figure is drawn by authors using “CorelDraw 14” software.

Modern *B. asiatica* grows on the southern slope of the Himalayas at altitudes ranging from 914 to 2450 m (Fig. 3 green range)^25^,^26^,^27^. The NLR (Nearest Living Relative) concept^28^, assumes that the fossil *B.* cf. *asiatica* and its nearest living species *B. asiatica* occupied similar or identical niches and lived at correspondingly similar altitudes. Normally such an assumption could not be justified but extensive analysis of fossil occurrences in Eurasia spanning the Miocene to present using the Co-Existence Approach^29^ indicate that the genus *Berberis* does not occur as an outlier (Utescher, T., personal communication August 2014, see the e-mail permission messages in SI) and thus can be regarded as a conservative genus. On this basis the altitudinal range of *B.* cf. *asiatica* is inferred to be similar to or the same as that of *B. asiatica*.

## Discussion

### Altitude of northern Tibet during the early Miocene

To correct for secular climate differences between the middle Miocene and the present we used both modelled lapse rates and enthalpy^30^. Assuming that the mid-Miocene was 2.89 °C warmer than now^20^,^30^,^31^ and taking the lapse rate to be 6.01 °C/1000 m^11^,^30^, the maximum altitude at which *Berberis asiatica* could have grown in the early Miocene was 481 m higher than present due solely to this climatic difference. Therefore, when *B.* cf. *asiatica* was flourishing in the early Miocene the palaeoaltitude of the Wudaoliang Basin could have been no more than 1395–2931 m (Fig. 3 pink range). It therefore follows that because our fossil was found at a present day elevation of 4611 m the Wudaoliang Basin must have been uplifted by 1680–3216 m (Fig. 3) in the last 17 Ma.

### The uplift of northern Tibet

In recent years, both geophysical modelling and palaeoaltimetry studies have generated different hypotheses concerning the uplift history of the northern Tibetan Plateau. Some studies suggest northern Tibet attained today’s elevation in the Miocene^17^ while the “far field deformation” hypothesis advocates northern Tibetan uplift preceding that of southern Tibet as early as the middle to late Eocene^32^. Other views such as the ‘stepwise model’ propose that northern Tibet only achieved today’s elevation after the Miocene^2^,^33^.

Our estimate for the Wudaoliang Basin suggests a palaeoelevation of 1395–2931 m during the early Miocene, which is close to an estimation for the same area based on δ^18^O values in late Eocene carbonate sediments^14^. This shows that the elevation of northern Tibet probably remained stable from the late Eocene to early Miocene but since then there has been a considerable uplift (~2000 m).

Our finding does not support earlier views that northern Tibet had reached or even exceeded its modern elevation before the Miocene^17^,^32^, but seems to be close to the predictions of stepwise uplift models, suggesting a significant uplift of northern Tibet since Miocene time^2^,^33^. Here the palaeoaltitude of northern Tibet estimated by using new independent biological evidence provides cross validation of results from geophysical models and geochemical evidence.

## Methods

The fossil leaf architecture characters were exposed by dégagement and examined under a stereomicroscope and an environmental scanning electron microscope. The leaf architecture is described following the terminology of the Leaf Architecture Working Group^24^. The comparative extant *Berberis* material came from the PE Herbarium of the Institute of Botany, Chinese Academy of Sciences and the G.B. Pant Institute of Himalayan Environment and Development, India. It was cleared in a 10% aqueous solution of NaOH.

### Palaeoaltimetry correction

Because the global climate has undergone a secular change in temperature of about 2.89 °C since the early Miocene^20^,^30^,^31^, a correction factor based on the palaeotemperature has to be applied when estimating palaeoaltitude. Assuming the Miocene lapse rate to be 6.01 °C/1000 m^30^, the corrected altitude is derived as follows:
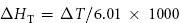
(Where ΔH_T_ is the correction in palaeoaltitude, and ΔT is the temperature difference between the Miocene and today).

## Author Contributions

Y.F.W. and C.S.L. conceived the ideas; M.Z., S.Q.W. and T.D. collected the fossils; B.S., Y.L.L. and Y.F.W. identified the fossil and analyzed the data; B.S. and Y.F.W. wrote the first draft of this manuscript; C.S.L., J.Y., J.F.L., T.D. and R.C.M. revised the draft versions. R.A.S. and D.K.F. rewrote some of the discussion and corrected the final manuscript.

## Additional Information

**How to cite this article**: Sun, B. *et al*. Early Miocene elevation in northern Tibet estimated by palaeobotanical evidence. *Sci. Rep.* **5**, 10379; doi: 10.1038/srep10379 (2015).

## Supplementary Material

Supplementary Information
