# Supplementary material for: Early Miocene elevation in northern Tibet estimated by palaeobotanical evidence
Source: Sci Rep. 2015 May 15;5:10379. doi: 10.1038/srep10379 (PMC4432560; doi:10.1038/srep10379)
Supplement: Supplementary Information [file srep10379-s1.pdf]

Supplementary Information

Early Miocene elevation in northern Tibet estimated by palaeobotanical evidence

Bin Sun[1, 7], Yu-Fei Wang[1]*, Cheng-Sen Li[1]*, Jian Yang[1], Jin-Feng Li[1], Ye-Liang Li[1, 6], Tao Deng[2], Shi-Qi Wang[2], Min Zhao[2], Robert A. Spicer[1, 3], David K. Ferguson[1, 4] & Rakesh C. Mehrotra [5]

[1]State Key Laboratory of Systematic and Evolutionary Botany, Institute of Botany, Chinese Academy of Sciences, Beijing 100093, China.

[2]Key Laboratory of Vertebrate Evolution and Human Origins, Institute of Vertebrate Palaeontology and Palaeoanthropology, Chinese Academy of Sciences, Beijing 100044, China.

[3]Environment, Earth and Ecosystems, Centre for Earth, Planetary, Space and Astronomical Research, The Open University, Milton Keynes, MK7 6AA, UK.

[4]Department of Palaeontology, University of Vienna, Althanstrasse 14, A-1090 Vienna, Austria.

[5]Birbal Sahni Institute of Palaeobotany, 53 University Road, Lucknow 226007, India

[6]Henan University of Traditional Chinese Medicine, No.1 Jinshui Road, Zhengzhou, Henan, 450008, China.

[7]University of Chinese Academy of Sciences, Beijing 100039, China.

*Correspondence to: Yu-Fei Wang, tel. +86 10 6283 6439, e-mail:wangyf@ibcas.ac.cn;

Cheng-Sen Li, tel. +86 10 6283 6436, e-mail: lics@ibcas.ac.cn.

Contents

Part 1. Supplementary Geologic setting

Part 2. Supplementary Systematics

Part 3. Supplementary Figures S1 - S3

Part 4. Supplementary Tables S1 - S2

Part 5. Supplementary Keys S1 - S2

Part 6. Supplementary References

Part 1. Supplementary Geologic setting.

According to the core data (Fig. 1), there are Quaternary hill-slope accumulations from 0.0–1.0 m depth followed by Wudaoliang Group lacustrine sediments from 1.0–151.3 m. Lithologically these consist of limestone, dolomitic limestone and marl, with mudstone of varying thickness. The occurrence of brown-red residual-slope accumulations with thin-bedded calcified fragments between 50.0–51.0 m denotes a hiatus in the lacustrine sediments. On either side of this hiatus there is a continuity of lacustrine sediments[18].

The dating of core ZK1 (Fig. 1) is calibrated as 24.1±0.6 Ma to 14.5±0.5 Ma, using climatostratigraphic methods, based on a comparison between the palaeoclimatic cycles recorded by carbon and oxygen isotope changes of lacustrine deposits[18] and the palaeoclimatic cycles recorded by the deep-sea oxygen isotope curve[19]. The ages at both top and bottom of the Wudaoliang Group calibrated in this way are in accordance with previous data from dating of Wudaoliang volcanics[20-22].

In northern Tibet, some late Cenozoic mafic volcanic rocks unconformably overlie the Wudaoliang group lacustrine sedimentary strata[18]. The basaltic andesite in the northern Tibetan Canmei Mountain area, overlying these Miocene lacustrine sedimentary strata, was dated with a whole-rock K-Ar age to 12.85±0.56 Ma ~ 14.51±0.23 Ma[21]. The normal faulting in the central Tibetan Shuang Hu Graben was dated as 13.5±1.4 Ma based on the Rb-Sr isotope data for muscovite, quartz, calcite, and whole rock[22]. These two results indicate that deposition in the Miocene ancient lake ended at about 13.5±1.4

Ma ~ 14.51 ±0.23 Ma.

The Wudaoliang group lacustrine sedimentary strata unconformably onlap the late Oligocene thrust systems, so the isotope data from the underlying tectonites and intrusive rock place important geochronological constraints on the maximum age of the Wudaoliang group[18]. Thrusting in the southern Kunlun Mountains and granite emplacement, caused by thrusting in Fenghuo Mountain, are inferred to have occurred at 28.8 Ma and 26.5 Ma respectively, based on ^{39}Ar – ^{40}Ar and U – Pb dating, which means that the Miocene ancient lake became established after 28.8 Ma ~ 26.5 Ma[20].

Comparisons of the coordinates and altitude of our fossil layer with those of core ZK1, enabled us to correlate the fossil layer with a depth of 55 m in the ZK1 drill hole, hence an age of ca. 17 Ma.

Part 2. Supplementary Systematics

Family: Berberidaceae

Genus: *Berberis* L.

Species: *Berberis* cf. *asiatica* Roxb. ex DC.

Description. Leaf simple, symmetrical, elliptic or obovate, about 2.3 cm in length, 0.8 cm in width; laminar L:W ratio ca. 3:1. Leaf margin dentate, with 4-5 spinose or setaceous teeth on each side; each tooth 0.2 mm in length, apical side concave, basal side convex; central principal vein of tooth straight, accessory veins converging with principal vein, tooth apex spinose. Leaf apex acute. Leaf base cuneate. Venation pinnate. Primary

vein thick, straight or slightly curved. Secondary veins with festooned brochidodromous venation, 3 pairs diverging from base to apex, looping with upper secondary veins. Secondary vein spacing increasing towards base. Inter secondary veins simple, fusing with secondary veins. Tertiary veins forming a randomly reticulate pattern, with individual veins diverging at inconsistent angles.

Comparison with fossil species of *Berberis*. Seventeen species of fossil *Berberis*, preserved as leaves, have been reported[23,34-43]. There are more than two significantly distinct differences in the leaf architecture of our fossil from those already described in the literature. Six fossil species are similar to the fossil specimen from Wudaoliang (Fig. 2 G–L). However, *B. teutonica* (Fig. 2 H), *B. poblana* (Fig. 2 J), and *B. longipetiolata* (Fig. 2 K) with either entire or densely toothed margin are distinguished from the fossil leaf, while the spinose *B. lycium* (Fig. 2 I) possesses 8-9 pairs of secondary veins, vs 3-4 pairs of secondary veins in our fossil. Both *B. huziokai* (Fig. 2 G) and *B. ahuehuetensis* (Fig. 2 L) have suprabasal veins, while the Wudaoliang fossil specimen has basal veins. The distinctions between the Wudaoliang specimen and other fossil species are listed in detail in Table S1 and a key is given in Supplementary Key 1.

Comparison with extant species of *Berberis*. The genus（ca. 500 extant species）with a South American-Old World disjunctive distribution is divided into two groups: Group Septentrionales (300 species having brochidodromous or semicraspedodromous venation) and Group Australes (200 species having actinodromous or acrodromous venation)[23]. The

former group with its brochidodromous or semicraspedodromous venation resembles the present fossil. Only 133 species within the group Septentrionales are similar in having elliptic or obovate leaves, and only 76 species of these 133 species have semicraspedodromous venation, of which 40 species have spinose or setaceous teeth, acute apex and cuneate base. Among these 40 species, only four species–*B. phanera, B. taronensis, B. chitria, B. asiatica*, have 2–8 teeth on each side. Therefore, these four species were selected for detailed comparison with the fossil *Berberis* from Wudaoliang (Fig. 2, C–F, and Fig. S1 E-H).

The comparison (see Table S2 and Key 2) indicates that the fossil is indistinguishable from *B. asiatica*, for the leaf architectural characters of the fossil are identical to those of *B. asiatica* except that no fossil veinlets are preserved. The other 3 species are clearly distinguishable from the Wudaoliang fossil, i.e., *B. phanera* with 6-8 pairs of secondary veins, differs from the fossil specimen with only 3-5 pairs. The fossil specimen has teeth on the distal 1/3 of the leaf, while *B. taronensis* has no tooth in this area. Moreover, the tooth's principal vein in *B. chirita* is eccentric, while that of the fossil is central.

Part 3. Supplementary Figures S1 - S3

Supplementary Figure S1

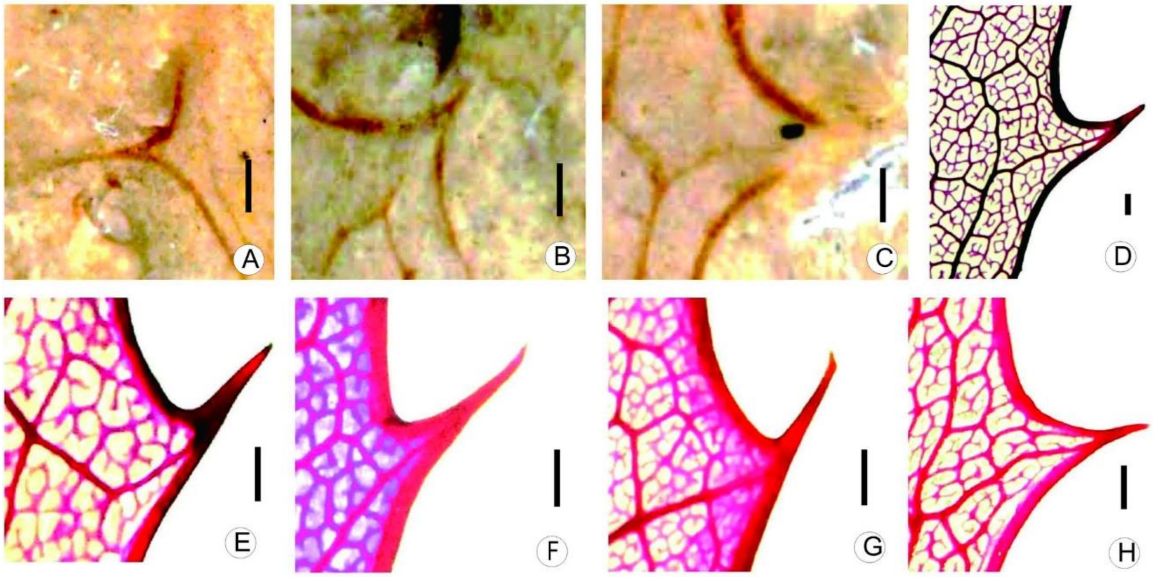

Figure S1. Details of tooth structures of *Berberis*, scale bar=1 mm

A–C. Fossil from Wudaoliang; D. *B. asiatica* E. *B. chitria*; F. *B. taronensis*;

G. *B. Phanera*; H. *B. purdomii*

Supplementary Figure S2

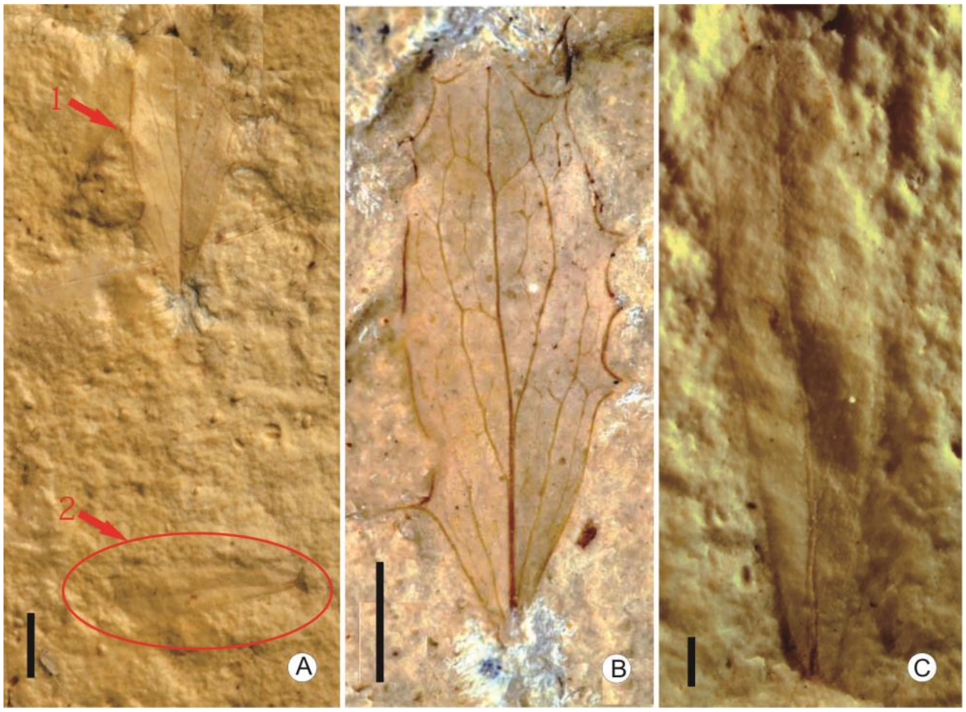

Figure S2. Two leaf impressions in one block

A. The block with two leaf impressions; B. The enlargement of 1 in A, *Berberis* cf. *asiatica*; C. The enlargement of 2 in A, another leaf impression in the same block

A, B, scale bars=5 mm; C, scale bar=1 mm

Supplementary Figure S3

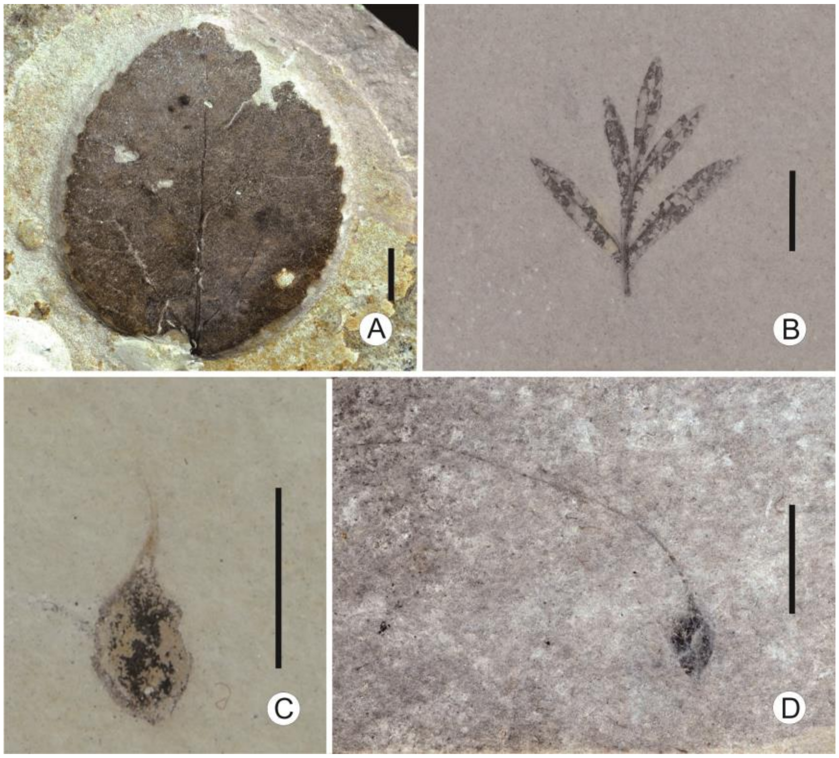

Figure S3. Fossil plants found from the Wudaoliang Formation, early Miocene, northern Tibet

A. A dicot leaf with simple teeth; B. A compound leaf; C, D. Grass-like fruits.

A, B, C, D, scale bars=1 cm

Part 4. Supplementary Tables S1 - S2

Supplementary Table S1. Morphological comparison with fossil species of *Berberis* (1)

	Fossil *Berberis* from Wudaoliang	*B. wuyunensis*	*B. lozanofolia*	*B. poblana*	*B. tepexiana*	*B. ahuehuetensis*	*B. hirsuta*	*B. coloradensis*	*B. lycium*
Shape	elliptic	oblong*	elliptic, obovate	elliptic, obovate	obovate*	obovate*	narrow oblong*	lanceolate*	linear-oblong*
Symmetry	symmetrical	symmetrical	symmetrical	symmetrical	symmetrical	symmetrical	symmetrical	symmetrical	symmetrical
Length (cm)	2.3	>3.5, ca. 4	0.9-1.6	1.3	0.85	3.3	2.0	1.7-2.2	2.54
Width (cm)	8	1.3	0.4-0.5	0.5	0.6	1.2	0.5	0.3-0.5	0.76
Length/width ratio	3:1	3:1	2.2-3.2:1	2.6:1	1.4:1	2.7:1	4:1	5:1	3.3:1
Petiole	no data	no data	short	short	short	short	short	short	short
Apex	acute	rounded*	acute, narrowly rounded	rounded, mucronate*	rounded*	narrowly rounded*	rounded*	acute, mucronate	acute
Base	cuneate	no data	cuneate	cuneate	cuneate	cuneate	rounded*	cuneate	cuneate
Margin	dentate, with 4-5 spinose or setaceous teeth on each side	serrate, with densely spinose teeth*	entire, occasionally with single tooth*	entire*	simple serrate, with densely spinose teeth*	serrate, with large teeth	entire*	serrate, with large teeth	serrate
Venation	pinnate	pinnate	acrodromous*	acrodromous*	acrodromous*	pinnate	pinnate	acrodromous *	pinnate
Primary veins	thick, straight	thick, straight	thick, straight	thick, sinuous*	thick, straight	thick, slightly curved*	thick, straight	thick, straight	thick, straight
Secondary veins	semicraspedodromous,thick, diverging steeply, vein spacing increasing towards base	brochidodromous, diverging steeply, vein spacing increasing towards base	fusing with the principal*, vein spacing increasing towards base	fusing with the principal*, asymmetric, vein spacing increasing towards base	connecting mid-vein* and principal veins, vein spacing increasing towards base	brochidodromous, diverging steeply, vein spacing irregular	brochidodromous, diverging at high angles (80°)*	fusing with the secondary veins*, vein spacing increasing towards base	semi-crasped - odromous, vein spacing uniform*
Intersecondary veins	fusing with secondary veins	fusing with secondary veins	no data	no data	no data	no data	no data	no data	no data
Tertiary veins	random reticulate	random reticulate	reticulate	reticulate	reticulate	no data	irregularly looping*	no data	reticulate
Higher order veins		areoles imperfect, veinlets three times branched	no data	no data	no data	no data	veinlets branched	no data	no data
Locality	Tibet, China	Heilongjiang, China	Puebla, Mexico	Puebla, Mexico	Puebla, Mexico	Puebla, Mexico	Idaho, USA	Colorado, USA	Kashmir, India
Age	middle Miocene	Palaeocene	Oligocene	Oligocene	Oligocene	Oligocene	Oligocene	Oligocene	Pleistocene
Reference	this paper	Li *et al.* 2010[23]	Ramirez & Cevallos-Ferriz, 2000[35]	Ramirez & Cevallos-Ferriz, 2000[35]	Ramirez & Cevallos-Ferriz, 2000[35]	Ramirez & Cevallos-Ferriz, 2000[35]	Axelrod, 1998[36]	Axelrod, 1987[37]	Puri, 1946[34]

Supplementary Table S1. Morphological comparison with fossil species of *Berberis* (2)

	B. huziokai	*B. longipetiolata*	*B. bursukensis*	*B. andreanszkyi*	*B. kymeana*	*B. teutonica*	*B. berberidifolia*	*B. goinai*	*B. cf. mougeoti*
**Shape**	narrowly obovate*	elliptic*	elliptic*	lanceolate*	linear-lanceolate*	obovate*	obovate*	obovate*	elliptic*
Symmetry	symmetrical	symmetrical	symmetrical	symmetrical	symmetrical	symmetrical	asymmetrical*	symmetrical	asymmetrical*
Length (cm)	4.5	3.4	2.5	no data	2.9-11	3.5-5.0	1.1	5.5	4
Width (cm)	1	1.3	1-1.2	1-1.3	0.4-0.9	1.5-3.5	0.7	2.8	2.5
Length/width ratio	4.5:1	2.6:1	2-2.5:1	no data	7.2-12.2:1	1.6-2.3:1	1.6:1	2:1	1.6:1
**Petiole**	short	short	short	no data	short	subsessile	sessile	sessile	sessile
**Apex**	rounded*	no data	obtuse*	acuminate*	acute	acuminate*	obtuse*	rounded*	obtuse*
**Base**	cuneate, decurrent*	cuneate	decurrent*	no data	narrowly cuneate	rounded*	decurrent*	decurrent*	cuneate
**Margin**	serrate	simple serrate	entire, occasionally with one tooth*	simple serrate	small, widely spaced* with tiny spinose teeth	serrate	serrate	serrate	serrate
**Venation**	pinnate	pinnate	pinnate	pinnate	pinnate	pinnate	pinnate	pinnate	pinnate
Primary veins	thin, nearly straight	thick, slightly curved*	thick, slightly curved*	thick ,straight	thick, slightly bent*	thick, straight	thick, straight	thick, straight	thick, straight
Secondary veins	semicraspedodromous, vein spacing increasing towards base*	brochidodromous, vein spacing irregular*	brochidodromous, vein spacing irregular*	semicraspedodromous, looping along the margin, vein spacing uniform*	brochidodromous,* semicraspedodromous, joined in irregular areoles, vein spacing uniform	semicraspedodro-mous, vein spacing increasing towards base	brochidodro-mous, vein spacing irregular*	reticulodromous, secondary veins branched, vein spacing irregular*	reticulodromous, secondary veins branched, vein spacing irregular*
Inter-secondary veins	subparallel to secondary veins	no data	no data	sinuous and irregular	no data	subparallel to secondary veins	sinuous and irregular	no data	no data
Tertiary veins	thin, random reticulate	no data	random reticulate	sinuous and irregular*	oblique to the secondary veins*	random reticulate	random reticulate	random reticulate	random reticulate
Higher order veins	no data	no data	no data	areoles isometric	areoles imperfect	no data	no data	no data	no data
**Locality**	Hokkaido, Japan	Stamata, Russia	Stamata, Russia	Hungary	Aix-en-Provence, France, Kymi, Greece	Styria, Austria	Czech Republic, Bulgaria	Romania	Romania
**Age**	Miocene	Miocene	Miocene	middle Miocene, upper Oligocene	middle Miocene, upper Oligocene	Miocene	Miocene	Miocene	Miocene
**Reference**	Tanai & Suzuki 1963[38]	Takhtajan, 1974[39]	Takhtajan, 1974[39]	Kvaček & Erdei, 2001[40]	Kvacek & Erdei, 2001[40]	Kovar-Eder *et al.*, 2004[41]	Kvaček *et al.*, 2004[42]	Givulescu, 1990[43]	Givulescu, 1990[43]

Note: * indicating differences between fossil *Berberis* from Wudaoliang and other species.

Supplementary Table 2. Morphological comparison with extant species of *Berberis*

	Fossil *Berberis* from Wudaoliang	*B. asiatica*	*B.chitria*	*B. phanera*	B.*taronesis*
Shape	elliptic, obovate	elliptic, obovate	elliptic, obovate	long elliptic, obovate	narrow elliptic, lanceolate
Symmetry	nearly symmetrical	symmetrical	symmetrical	symmetrical	symmetrical
Length (cm)	2.3	3.5-6.5	2.5	4.5-7	2.5-6
Width (cm)	0.8	1.2-2.2	1	1.2-1.8	0.6-1.5
Length/width ratio	3:1	3:1	2.5:1	4:1	4:1
Petiole (cm)	nearly absent	nearly absent	nearly absent	4-5	nearly absent
Apex	acute	acute	acute	acute	acute
Base	cuneate	cuneate	cuneate	cuneate	cuneate
Margin	dentate, with 4-5 spinose or setaceous teeth on each side	slightly revolute, 5-6 spinose or setaceous teeth on each side	slightly revolute, 2-4 spinose or setaceous teeth on each side	slightly revolute, 7-12 spinose or setaceous teeth on each side	slightly revolute, 2-7 spinose or setaceous teeth on each side
Tooth shape (apical side/basal side)	concave/straight	concave/straight	concave/straight	concave/straight	concave/straight
Tooth sinus	rounded	rounded	rounded	rounded	rounded
Tooth principal vein	direct,central	direct,central	deflected,eccentric	direct,central	direct,central
Tooth accessory vein	connivent	connivent	connivent	connivent	connivent
Venation	pinnate	pinnate	pinnate	pinnate	pinnate
Primary vein	thick, sinuous	thick, sinuous	thick, sinuous	thick, sinuous	thick, sinuous
Secondary veins	semicraspedodromous,thick, pairs less than 6	brochidodromous,thick, pairs less than 6	brochidodromous,thick, pairs less than 6	brochidodromous,thick, pairs less than 6	brochidodromous,thick, pairs more than 6
Intersecondary veins	fusing with secondary veins	fusing with secondary veins	fusing with secondary veins	fusing with secondary veins	fusing with secondary veins
Tertiary veins	random reticulate	random reticulate	random reticulate	random reticulate	random reticulate
Marginal veins	no data	incomplete	incomplete	incomplete	incomplete
Locality	Tibet,China	India and Pakistan	India and Pakistan	Yunnan and Sichuan, China	Yunnan and Tibet, China
Elevation (m)		914-2286	1800-2700	1800-4000	2030-2600

Part 5. Supplementary Keys S1 - S2

Supplementary Key S1. Key to fossil species of *Berberis*

1. Venation acrodromous...2

2. Margin entire or occasionally with single tooth..3

3. Leaf apex acute or narrowly rounded..*B. lozanofolia*

3. Leaf apex rounded with mucronate tip..*B. poblana*

2. Margin with spinose teeth...4

4. Lamina obovate, apex rounded..*B. tepexiana*

4. Lamina lanceolate， apex acute..*B. coloradensis*

1. Venation pinnate...5

5. Margin entire or occasionally with single tooth..6

6. Base and apex rounded, secondary veins diverging at right angles..................*B.hirsuta*

6. Base cuneate and decurrent, apex obtuse, secondary veins diverging at acute angles ....*B. bursukensis*

5. Margin with spinose teeth...7

7. Lamina asymmetrical...8

8. Secondary veins semicraspedodromous..*B. berberidifolia*

8. Secondary veins brochidodromous...*B.* cf. *mougeoti*

7. Lamina symmetrical...9

9. Secondary vein spacing uniform..10

10. Leaf apex acuminate...*B. andreanszkyi*

10. Leaf apex acute...11

11. Lamina linear-oblong, lamina L: W ratio 3.3:1.........................*B. lycium*

11. Lamina linear-lanceolate, lamina L: W ratio 7.2:1................*B. kymeana*

9. Secondary vein spacing irregular or increasing towards base............................12

12. Secondary vein spacing irregular...13

13. Secondary veins semicraspedodromous.............................*B. teutonica*

13. Secondary veins camptodromous...14

14. Secondary veins reticulodromous...................................*B. goinai*

14. Secondary veins brochidodromous...15

15. Lamina obovate..*B. ahuehuetensis*

15. Lamina elliptic..*B. longipetiolata*

12. Secondary vein spacing increasing towards base......................................16

16. Margin with densely spinose teeth…………………………..…..*B. wuyunensis*

16. Margin with spinose or setaceous teeth..17

17. Secondary veins semicraspedodromous and suprabasal...................

....*B. huziokai*

17. Secondary veins brochidodromous and basal…………………….…………….

……………………………………………………..….....Fossil *Berberis* from Wudaoliang

Part 5. Supplementary Keys S1 - S2

Supplementary Key S2. Key to fossil *Berberis* from Wudaoliang and its related living species

1. Margin with thin spinose teeth, evenly distributed on each side ……………..…………………….2

 2. Primary veins of teeth eccentric………………………………………………………………….……*B. chitria*

 2. Primary veins of teeth central…………………………….……………………………..……………………….…3

  3. Pairs of secondary veins more than 6…………………………….………………….…*B. taronesis*

  3. Pairs of secondary veins fewer than 6…………………....*B. asiatica*, fossil *Berberis* from Wudaoliang

1. Margin with thin spinose teeth, unevenly distributed on each side………………………………..4

 4. Primary veins of teeth are central with 3-5 spinose teeth on the proximal 2/3 of the leaf…………………………………………………………………………………….….…………………..*B. phanera*

 4. Primary veins of teeth are eccentric, margin rarely with spinose teeth, 1-2 or no teeth on each side………………………………………………………………………..…..………….*B. purdomii*

Part 6. Supplementary References

34. Puri, G. S. Some fossil leaves of *Berberis* from the Karewas of Kashmir. *J. Indian Bot. Soc*. **25,** 177-183 (1946).

35. Ramirez, J. L. & Cevallos-Ferriz, S. R. S. Leaves of Berberidaceae (*Berberis* and *Mahonia*) from Oligocene sediments, near Tepexi de Rodriguez, Puebla. *Rev. Palaeobot. Palynol*. **110,** 247–257 (2010).

36. Axelrod, D. I. The Oligocene Haynes Creek flora of Eastern Idaho. *Univ. Calif. Publ.Geol. Sci*. **143,** 1–235 (1998).

37. Axelrod, D. I. The late Oligocene Creede flora, Colorado. *Univ. Calif. Publ. Geol. Sci*. **130,** 1–235 (1987).

38. Tanai, T. & Suzuki, N. in *Miocene floras of southwestern Hokkaido, Japan. In: Chaney, R. W., Tanai, T. (Eds.), Tertiary Floras of Japan: Volume 2. Miocene floras* 9–152 (The Collaborating Association to Commemorate the 80th Anniversary of the Geological Survey of Japan, Tokyo, 1963).

39. Takhtajan, A. *Berberidaceae.* In: *Takhtajan, A. (Ed.), Magnoliophyta Fossilis U.R.S.S., volume 1* 109–111Nauka (Leningrad, in Russian, 1974)

40. Kvaček, Z. & Erdei, B. Putative proteaceous elements of the Lomatites-type reinterpreted as new Berberis of the European Tertiary. *Plant Syst. Evol*. **226,** 1–12 (2001).

41. Kovar-Eder, J., Kvaček, Z. & Ströbitzer-Hermann, M. The Miocene Flora of Parschlug (Styria, Austria)—Revision and Synthesis. *Ann. Naturhist. Mus. Wien* **105A,** 45–459 (2004).

42. Kvaček, Z., Dvořák, Z., Mach, K. & Sakala, J. Třetihorní rostliny severočeské hnědouhelné pánve. Severočeské doly a. s., Chomutov Granit, s. r. o., Praha (2004).

43. Givulescu, R. in *Flora fosilă a Miocenului Superior de Chiuzbaia. Ed.* (Acad. Române, Bucureşti, 1990).
